# *Plectorhinchus makranensis* (Teleostei, Haemulidae), a new species of sweetlips from the Persian Gulf and the Gulf of Oman

**DOI:** 10.3897/zookeys.980.50934

**Published:** 2020-10-28

**Authors:** Ehsan Damadi, Faezeh Yazdani Moghaddam, Fereshteh Ghassemzadeh, Mehdi Ghanbarifardi

**Affiliations:** 1 Department of Biology, Faculty of Sciences, University of Ferdowsi, Mashhad, Iran University of Ferdowsi Mashhad Iran; 2 Zoological Innovations Research Department, Institute of Applied Zoology, Faculty of Science, Ferdowsi University of Mashhad, Mashhad, Iran Ferdowsi University of Mashhad Mashhad Iran; 3 Department of Biology, Faculty of Science, University of Sistan and Baluchestan, Zahedan, Iran University of Sistan and Baluchestan Zahedan Iran

**Keywords:** Haemulidae, morphology, mtDNA, Northwest Indian Ocean, phylogenetic relationships, *
Plectorhinchus
*

## Abstract

*Plectorhinchus makranensis***sp. nov**. is described on the basis of 16 specimens from the Persian Gulf and Gulf of Oman, in the Northwest Indian Ocean. The new species can be distinguished from congeners by a combination of dorsal fin rays XII, 18–20, pectoral-fin rays 16–17, tubed lateral-line scales 55–57, gill rakers count (10–12 on the upper limb and 16–17 on the lower limb), 17–18 scales between the lateral line and the first anal-fin spine, 30–31 circumpeduncular scale rows and color pattern. *Plectorhinchus makranensis***sp. nov.** is distinguished from *P. schotaf* by having the posterior margin of the opercular membrane grey (vs. red in *P. schotaf*), fewer circumpeduncular scale rows, and a shorter base of the soft portion of the dorsal fin, 27.6–29.4% of standard length (SL) (vs. 31–32.3% of SL in *P. schotaf*). The new species resembles *P. sordidus* but is differentiated from it by having more gill rakers, a smaller orbit diameter 27.5–32.1% of head length (HL) (vs. 35.5–37.2% of HL in *P. sordidus*), a longer caudal peduncle 19.2–21.3% of SL (vs. 17.1–17.9% of SL in *P. sordidus*), and the first to third pectoral-fin rays light gray (vs. dark gray in *P. sordidus*). The new species can also be distinguished from the other species, including *P. schotaf* and *P. sordidus*, based on COI and Cyt *b* molecular markers. The phylogenetic position of this new species indicates that it is a sister taxon of *P. schotaf*.

## Introduction

Haemulidae Gill, 1885, is one of the 10 largest families of the order Perciformes, with 19 genera and 136 species. Almost half of the world’s haemulid species belong to the genera *Plectorhinchus* and *Pomadasys* (Nelson et al. 2016; Fricke et al. 2019). The genus *Plectorhinchus* Jordan & Thompson, 1912 (Perciformes: Haemulidae) is commonly called sweetlips and includes fish with commercial importance in the Indo-west Pacific Ocean (Liang et al. 2016; Froese and Pauly 2019). Many species of this genus have markedly different color patterns, morphological characteristics, and ecological characteristics (Randall et al. 1997; Johnson et al. 2001; McKay 2001). The genus *Plectorhinchus* is widely distributed in the Indo-Pacific and eastern Atlantic (Tavera et al. 2012; Froese and Pauly 2019) and encompasses 31 valid species worldwide (Fricke et al. 2019). Seven species of *Plectorhinchus* are known to exist in the Gulf of Oman: *Plectorhinchus flavomaculatus* (Cuvier 1830), *P. gaterinus* (Forsskål, 1775), *P. gibbosus* (Lacepède, 1802), *P. pictus* (Tortonese, 1936), *P. playfairi* (Pellegrin, 1914), *P. schotaf* (Forsskål, 1775) and *P. sordidus* (Klunzinger, 1870) (Randall 1995); however, Carpenter et al. (1997) and Bishop (2003) reported only three species in the Persian Gulf (*P. gaterinus*, *P. pictus* and *P. sordidus*). Phylogenetic relationships of the genus *Plectorhinchus* have previously been investigated (Sanciangco et al. 2011; Liang et al. 2016; Tavera et al. 2018). Molecular phylogenetic investigations of ichthyofauna are rare in the studied area (Asgharian et al. 2011; Ghanbarifardi et al. 2016; Polgar et al. 2017; Rabaoui et al. 2019). Asgharian et al. (2011) and Johnson and Wilmer (2015) reported two specimens of *P. schotaf* in the Persian Gulf represented two genetic lineages, based on the COI gene.

The aims of our study are to use two molecular markers (COI, Cyt *b*) and morphological characters to confirm the existence of two lineages proposed by the other authors and to describe a new species of *Plectorhinchus* collected from the Gulf of Oman and the Persian Gulf.

## Materials and methods

### Sampling and material examined

In the present study 16 specimens of *Plectorhinchus* spp. and 10 specimens of *P. schotaf* were collected from six localities (Gulf of Oman: Beris, Tis, Pozm, Jask; Persian Gulf: Kangan, Hendijan) by gill netting in the time period from August 2017 to June 2018 (Fig﻿. 1). All specimens are deposited in the Zoological Museum, Ferdowsi University of Mashhad (ZMFUM), Iran. Muscle tissue of the specimens were taken and fixed in absolute ethanol for molecular analysis and the specimens were stored at -20 °C for later morphological study.

**Figure 1.**
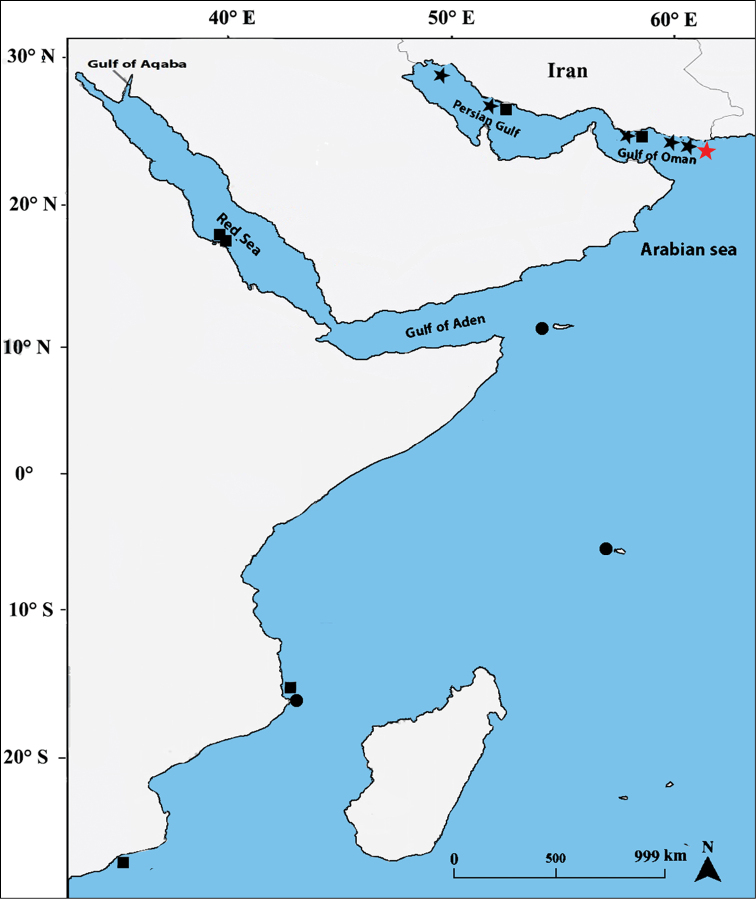
Sampling localities and distribution of *Plectorhinchus makranensis* sp. nov. (stars), *P. schotaf* (squares) and *P. sordidus* (circles) across the Western Indian Ocean. Red star: Beris (type locality).

### DNA extraction, PCR and sequencing

Genomic DNA was extracted from 10 specimens of *Plectorhinchus*, including six *Plectorhinchus makranensis* sp. nov. and four *P. schotaf*, following the GeNet Bio kit protocol. Sequences were amplified by PCR using the following primer pairs: cytochrome oxidase subunit 1 (CO1LBC_F: 5’ TCAACYAATCAYAAAGATATYGGCAC 3’; CO1HBC_R: 5’ ACTTCYGGGTGRCCRAARAATCA 3’) and Cytochrome b (GluF: 5’AACCACCGTTGTATTCAACTACAA3; ThrR: 5’ACCTCCGATCTTCGGATTACAAGACCG3), following Ward et al. (2005) and Machordom and Doadrio (2001), respectively. PCR conditions for the COI gene included: initial denaturation 94 °C, 1 min then 30 cycles at 95 °C for 30 s, 52 °C for 45 s, and 72 °C for 1 min, followed by a final extension at 72 °C for 5 min. All amplification conditions were similar the COI gene except for the annealing temperature (54 °C) and the number cycles (35) for the Cyt *b* gene. The quality of PCR products was determined by running them on 1.5% agarose gels in 0.5X TBE buffer. The products were sent to Microsynth Company (Switzerland) for sequencing. We analysed a total of 64 sequences *of Plectorhinchus* species, including 36 for COI and 28 for Cyt *b.* Sequences of *Pomadasys maculatus* (Bloch, 1793) and *Haemulon aurolineatum* Cuvier, 1830 were used as outgroups.

### Molecular data analyses

All sequence alignments were performed using the MAFFT algorithm. The pairwise DNA sequence differences within and between species of *Plectorhinchus* were calculated with MEGA 7.0.9 (Kumar et al. 2016) based on the Kimura two-parameter (K2P) model. The best-fit nucleotide substitution models were determined by jModelTest (Posada 2008) for each gene and combination of two genes. Based on Akaike information criterion (AIC), the preferred model for the two molecular markers was TVM + I + G. Analyses of phylogenetic relationships were performed for each gene and combination of two genes (Cyt *b* + COI) using maximum likelihood (ML) and Bayesian inference (BI). ML analysis as implemented in RAxML 7.2.6 (Stamatakis 2014) with 10,000 bootstrap replicates. BI analysis was run for 30,000,000 generations in MrBayes 3·1·2 (Ronquist et al. 2012) with two independent runs of four Markov Chain Monte Carlo (MCMC). The first 25% of the trees were excluded as burn-in and remaining trees sampling were used to compute a 50% majority rule consensus tree. The resulting phylogenetic trees from ML and BI analyses were edited using FigTree v.1.4.4. Additionally, we used from two different approaches for species delimitation including the Automatic Barcode Gap Discovery (ABGD) (Puillandre et al. 2012) and Bayesian Poisson Tree Process (bPTP) (Zhang et al. 2013). The ABGD method based on the COI gene was performed on web http://wwwabi.snv.jussieu.fr/html, under the Kimura (K80) model with the default parameters of Pmin = 0.001 to Pmax = 0.1, steps = 10, X (relative gap width) = 1.5, Nb bins = 20. The bPTP approach used the best ML tree, which was run on the web server (http://species.h-its.org/ptp). This analysis was processed with 500,000 MCMC generations and 25% of burn-in.

### Morphological analysis

We used Johnson and Wilmer (2015) for morphometric and meristic characteristics which consisted of 23 morphometric and seven meristic features. The univariate and multivariate analysis were run in SPSS v.16 (SPSS Inc., Chicago IL) and PAST v. 4.03 (Hammer 2020). We assessed the normally distributed parametric data using the Shapiro-Wilk test. The morphometric characters were divided by standard length (SL) and head length (HL) to remove the size effect from the dataset. The univariate Analysis of Variance (ANOVA) was performed for morphometric characters to evaluate the significance of phenotypic differences between species. The principal components analyses (PCA) was used for multivariate analyses to characterize the morphological variation among species.

## Results

### Molecular analysis

**Figure 2.**
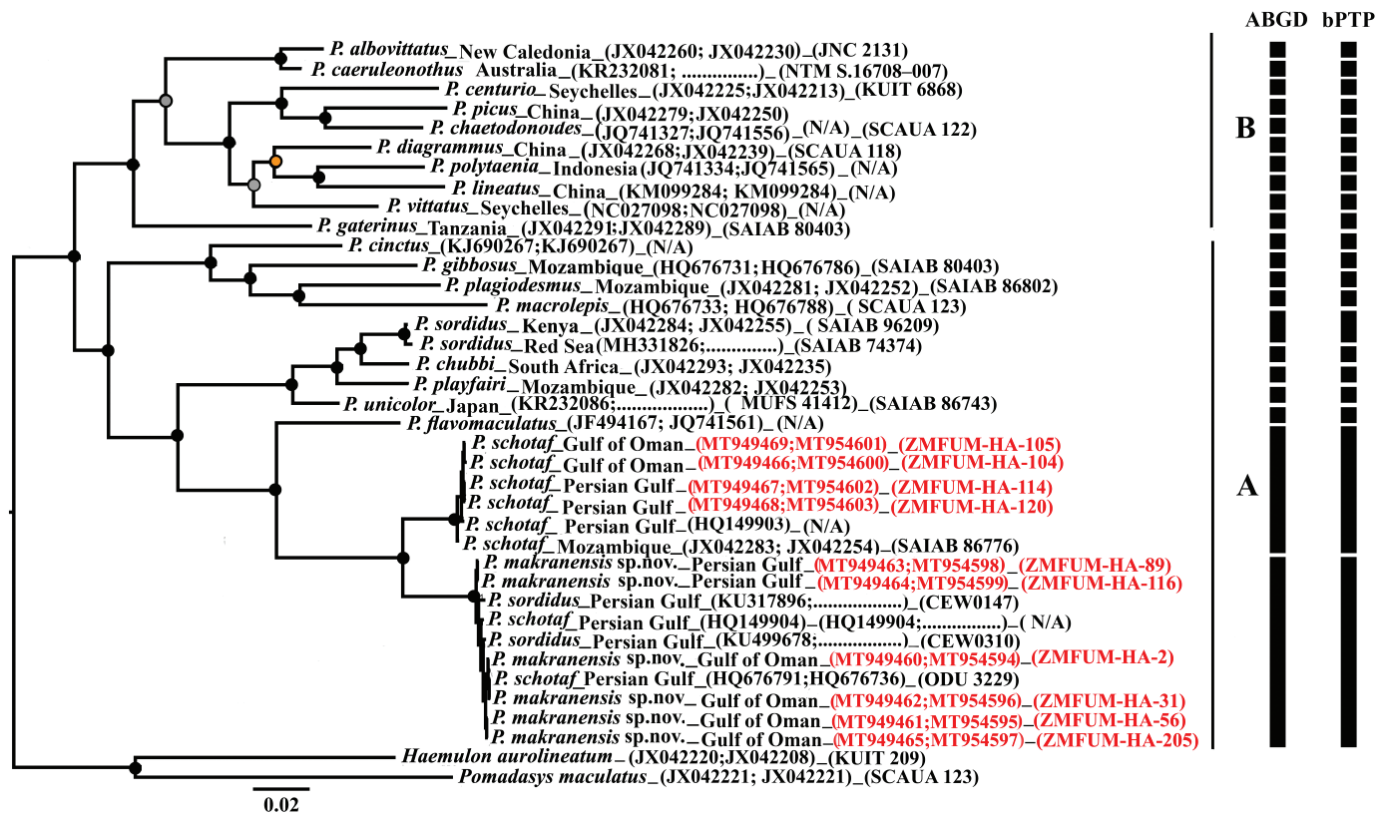
Molecular phylogenetic tree showing *P. makranensis* sp. nov. and other congeners based on two mitochondrial genes Cyt *b* and COI (total length 1672 bp). Supporting values of nodes: black circles (ML bootstrap BP ≥ 70% and BI probability PP ≥ 0.95), orange circles on nodes support by ML (BP ≥ 70%) but not by BI (PP ≥ 0.95), gray circles on nodes support by BI (PP ≥ 0.95) but not by ML (BP ≥ 70%). Bar at the side of the tree represents the results of the analyses of species delimitation. Red font: sequences of this study, COI (first), Cyt *b* (second).

This study used sequence data from 21 species of Haemulidae with two outgroups (38 samples) (Fig. 2). The combined dataset included 1672 bp (Cyt *b*: 1055, COI: 617), of which 629 bp (Cyt *b*: 450, COI: 179) were variable and 413 bp (Cyt *b*: 244, COI: 169) were parsimony-informative. Both ML and BI analyses yielded highly congruent trees with difference only in levels of support. *Plectorhinchus* species formed two clades with high bootstrap and posterior probabilities, including: clade A (*P. cinctus*, *P. gibbosus*, *P. plagiodesmus*, *P. macrolepis*, *P. sordidus*, *P. playfairi*, *P. chubbi*, *P. unicolor*, *P. flavomaculatusP. schotaf* and *P. makranensis* sp. nov.), and clade B (*P. albovittatus*, *P. caeruleonothus*, *P. centurio*, *P. picus*, *P. chaetodonoides*, *P. diagrammus*, *P. polytaenia*, *P. lineatus*, *P. vittatus* and *P. gaterinus*) (Fig. 2). *Plectorhinchus makranensis* sp. nov. was found to be closely related (see below) with *P. schotaf* which together comprised the sister group of *P. flavomaculatus*. The new species formed a highly supported monophyletic clade with low intraspecific genetic diversity for both two mtDNA markers (COI and Cyt *b*) (Fig. 2, Suppl. material 1: Table S1). *Plectorhinchus makranensis* sp. nov. demonstrated minimum interspecific genetic divergence with *P. schotaf* (4.78% for Cyt *b*, and 5.11% for COI) (Suppl. material 1: Table S1). *Plectorhinchus makranensis* sp. nov. exhibited maximum interspecific genetic divergence with *P. lineatus* (18.65% for Cyt *b*) and with *P. gaterinus* (20.32% for COI). The ABGD analysis based on the COI gene defined 21 MOTUs a wide range of P values (0.0028–0.0077). The bPTP analysis estimated number of MOTUs identical to ABGD MOTUs using COI and Cyt *b* markers with the support value ranging from 0.86 (*P. caeruleonothus* cluster) to 0.99 (the most cluster). These analyses also recognized the new species and its closest congener as distinct species (Fig. 2).

### Morphological analysis

#### Plectorhinchus makranensis sp. nov.

Taxon classificationAnimaliaPerciformesHaemulidae

E8B808AA-8C8B-5E3C-9DE7-5888AE15530F

http://zoobank.org/EAF907D6-B2FB-4BA3-8162-59148206D62F

**Figure 3.**
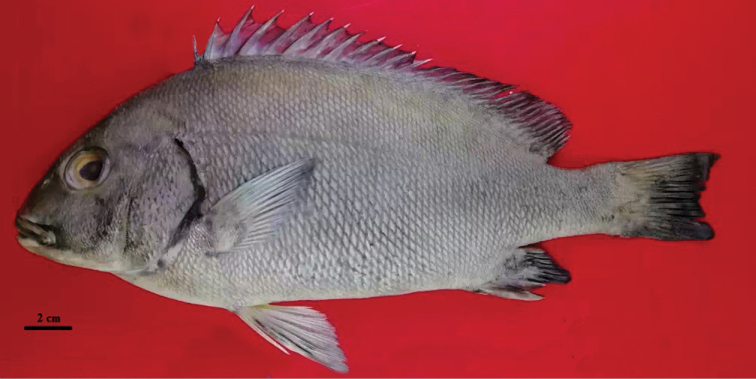
Holotype of *Plectorhinchus makranensis* sp. nov. ZMFUM-HA-56, 246.5 mm SL, Gulf of Oman: Beris coast (Photo by E. Damadi).

**Figure 4.**
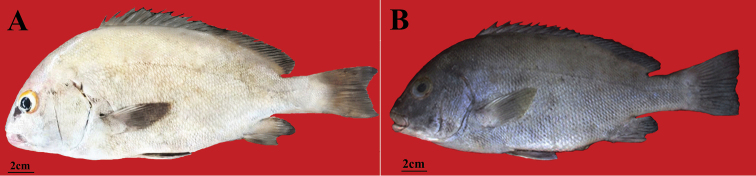
*Plectorhinchus makranensis* sp. nov. **a**ZMFUM-HA-205, paratype, 345.6 mm SL, Gulf of Oman: Tis coast, (Photo by E. Damadi) **b**ZMFUM-HA-75, paratype, 226.6 mm SL, Gulf of Oman: Tis coast, (Photo by E. Damadi).

**Figure 5.**
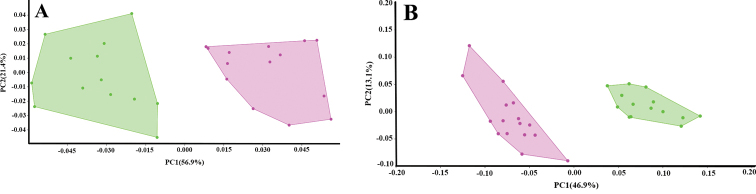
Principal components analysis (PCA) results for **A** meristic and **B** morphometric data of two species of *Plectorhinchus*: *Plectorhinchus makranensis* sp. nov. (pink); *P. schotaf* (green).

**Figure 6.**
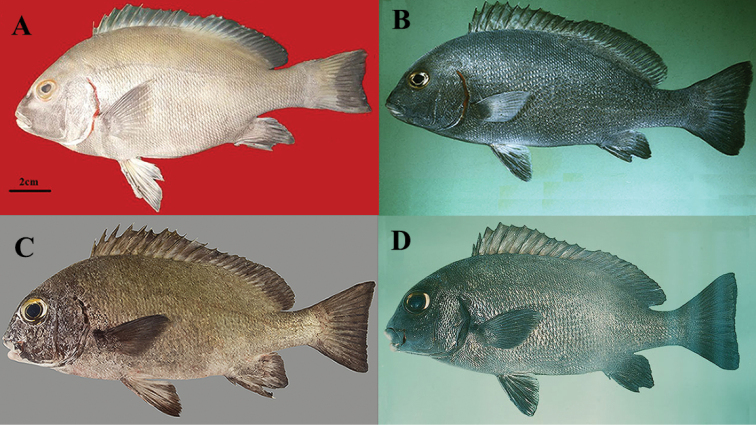
**A***Plectorhinchus schotaf*ZMFUM-HA-104, 268.9 mm SL, Gulf of Oman: Jask, (Photo by E. Damadi) **B***P. schotaf* BPBM 20766, 243 mm SL, Red Sea: Port Sudan, (Photo by J.E. Randall) **C***P. sordidus* SMF uncatalogued, 220 mm SL, Arabian Sea: Socotra, (Photo by S.V. Bogorodsky) **D***P. sordidus* BPBM 21379, 228 mm SL, Gulf of Oman. (Photo: J.E. Randall).

Figs 1 , 2 , 3 , 4 , 5 , 6 ; Suppl. material 1 : Table S1, Suppl. material 2: Table S2

##### Holotype.

(Fig. 3). ZMFUM-HA-56, 246.5 mm SL Type locality. Iran Gulf of Oman, Sistan and Baluchestan prov, Beris coast, 24°36'14"N, 61°45'77"E, depth 15 m, collected by Ehsan Damadi, 5 Aug 2017.

***Paratypes*** (*N* = 15). ZMFUM-HA-31, 141.2 mm SL, ZMFUM-HA-75, 226.6 mm SL, ZMFUM-HA-205, 345.6 mm SL, ZMFUM-HA-147, 295.3 mm SL and ZMFUM-HA-148 203.1 mm SL, all from the Gulf of Oman, Sistan and Baluchestan prov, Tis coast, 25°7'60"N, 61°6'28"E, depth 5 to 7 m, collected by E. Damadi, 10 Sep 2017; ZMFUM-HA-4, 246.5 mm SL and ZMFUM-HA-2, 228.5 mm SL, Gulf of Oman, Hormozgan prov, Pozm coast, 25°17'48"N, 59°59'12"E, depth 6 m, collected by M. Kahouri, 12 Oct 2017; ZMFUM-HA-44, 268.3 mm SL and ZMFUM-HA-45, 274.5 mm SL, Gulf of Oman, Hormozgan prov, Jask coast, 25°36'63"N, 57°45'37"E, depth 6 m, collected by H. Rahmani, 5 Nov 2017; ZMFUM-HA-74, 248.6 mm SL, and ZMFUM-HA-76, 301.1 mm SL, Gulf of Oman, Hormozgan prov, Jask coast, 25°28'43"N, 57°49'36"E, depth 6 to 9 m, collected by A. Amiri, 7 Dec 2017; ZMFUM-HA-78, 277.5 mm SL, ZMFUM-HA-87, 263.3 mm SL and ZMFUM-HA-116, 287.3 mm SL, Persian Gulf, Bushehr prov, Kangan Bandar, 27°33'20"N, 52° 7'09"E, depth 10 m, collected by H. Tangestani, 13 Apr 2018; ZMFUM-HA-89, 146.5 mm SL, Persian Gulf, Khuzestan prov, Henijan coast, 29°37'35"N, 49°59'79"E, depth 7 m, collected by H. Tangestani, 5 Jun 2018.

##### Comparative material.

***Plectorhinchus schotaf*** (*N* = 18): **Gulf of Oman**: ZMFUM-HA-103 to 106, four specimens, 268.9–290.9 mm, Jask, 5 Nov 2017, H. Rahmani; **Persian Gulf**: ZMFUM-HA-114 to 120, six specimens, 254.5–313 mm, Bushehr, 13 Apr 2018, H. Tangestani; **Red Sea**: BPBM 20355, 245 mm, Port Sudan, 9 Oct 1974, J.E. Randall; BPBM 20766, two specimens, 234–243 mm, Port Sudan, 14 Oct 1975, J.E. Randall; **Mozambique**: SAIAB 41668, four specimens, 100–121 mm, Inhaca, Sep 1948, J.L.B. & M.M. Smith; SAIAB 19796, 183 mm, Ibo, 8 Aug 1957, J.L.B. Smith.

***Plectorhinchus sordidus*** (*N* = 2): **Seychelles**: BPBM 21661, 230 mm, Caiman Rocks, 7 Jun. 1977, J.E. Randall; **Mozambique**: SAIAB 41668, 81 mm, 1 Sep 1948, J.L.B. and M.M. Smith.

##### Diagnosis.

*Plectorhinchus makranensis* sp. nov. can be distinguished from other congeners by the following combination of features: (1) meristic characters: dorsal fin rays XII, 18–20; gill rakers 10–12 + 16–17 (26–29); tubed lateral-line scales 55–57; transverse scale rows above lateral line 10–11; transverse scale rows below lateral line 17–18; circumpeduncular scales 30–31; (2) morphometric characters: base of soft portion of dorsal fin 27.6–29.4% of SL; orbit diameter 25.5–30.1% of HL; caudal peduncle length 19.2–21.3% of SL; (3) Color pattern: head and body unicolor without markings, the posterior part of the opercular membrane grey; uppermost first to third pectoral-fin rays light grey.

##### Description.

Meristic data and morphometric data are given in Suppl. material 2: Table S2. Dorsal-fin rays XII, 18–20 (modally 19), all soft rays branched except the first; anal-fin rays III, 7–8 (rarely 8), all soft rays branched; pectoral-fin rays 16–17 (modally 16), first and second rays unbranched; pelvic rays fin I, 5, all branched; caudal fin with 9 dorsal and 7 ventral rays (total = 16), uppermost and lowermost unbranched; tubed lateral-line scales 55–57 (modally 56); scales above lateral line to the base of the first dorsal-fin spine 10–11 (modally 10); scales below lateral line to first anal-fin spine 17–18 (modally 17); circumpeduncular scales 30–31 (modally 30); gill rakers on first arch small, 10–12 on upper limb (modally 12) and 16–17 on lower limb (modally 17); branchiostegal rays 7; preopercle with 31–36 serrae.

Body elongate, moderate deep, its depth 2.8–3.4 in SL, compressed laterally and covered with ctenoid scales; scales on the middle of the body largest; lateral line extends slightly as smaller scales onto the caudal-fin base; scales present on suborbital; snout and chin without scales; predorsal scales extending through interorbital. Head moderately large, head length 3.4–3.7 in SL, upper profile convex; mouth moderately small and terminal, lips fleshy, upper jaw protruding slightly beyond the lower jaw; nostrils small, posterior nostril half diameter of anterior nostril, anterior nostril on horizontal line through the lower margin of eye; orbit diameter 3.3–3.9 in HL; three pores on each side of the chin, but no pit; teeth cardiform, approximately 2 rows laterally and 5 rows anteriorly in the upper jaw, approximately 2 rows laterally and 6 rows anteriorly in lower jaw, approximately 20–24 teeth in the upper jaw on each side and approximately 16–18 in the lower jaw on each side, palatine and vomer without teeth. Opercle with a single, exposed, short and weak spine; preopercle slightly concave and serrate, including few serrae on the posteroventral margin.

Origin of dorsal fin above the pectoral-fin base, first spine shortest, fifth spine longest, first dorsal-fin spine about 1.2 length of fifth, first spine 6.4 (6.1–6.5) in HL, fifth spine 2.6 (2.2–2.7) in HL, 6^th^ and 7^th^ soft dorsal-fin ray longest, 6^th^ and 7^th^ 3.6 in HL, 18^th^ to 20^th^ soft dorsal-fin ray shortest, its length 9.6–9.8 in HL, base of soft portion of dorsal fin 1.1 in base of the spinous portion; anal fin short, with somewhat rounded posterior margin, origin below base of 7^th^ soft dorsal-fin ray, second spine longest, first ray is the longest, anal-fin length 2.5 (2.3–2.6) in HL; posterior margin of caudal fin slightly emarginate, caudal-fin length 1.7–1.8 in HL; pectoral fin reaching vertical between bases of seventh and eighth dorsal-fin spines, pectoral-fin length 1.4–1.5 in HL. Origin of pelvic fins behind pectoral-fin base, its tip reaching vertical at ninth dorsal-fin spine, second ray longest, pelvic-fin length 1.4–1.5 in HL.

##### Color pattern in preservative.

(***Holotype***: Fig. 3). Head and body steel grey; head and edge of fins slightly darker than the rest of the body; posterior part of opercular membrane dark grey; lips grey; ventral part of body including underside of head and belly to lower part of caudal peduncle white; iris yellow.

##### Color in fresh.

(paratypes: Fig. 4). Body silver-grey; all fins dark grey; pectoral-fin base light grey; uppermost first to third pectoral-fin rays light gray; orbital margin orange; iris grey; ventral part of body including subopercle, chest and pectoral-fin margin opaque white; lips and chin pink-grey; posterior part of opercular membrane grey.

##### Distribution and habitat.

The new species has been observed at six localities along the coast of the Gulf of Oman and the Persian Gulf in the Northwest Indian Ocean. Abundance was greater in the Gulf of Oman compared to the Persian Gulf. All specimens have been collected from shallow rocky and coral areas. Other species of this family which occur sympatrically at the type locality (Beris coast) with *Plectorhinchus makranensis* sp. nov. include: *Diagramma pictum*, *Plectorhinchus pictus*, *Pomadasys kaakan*, *P. maculatus* and *P. stridens*.

##### Etymology.

The species name is derived from the Makran coast and refers to the coastal land in southeastern Iran and southwestern Pakistan, north of the Gulf of Oman.

##### Multivariate analysis.

The first two Principal Components (PCs) of the meristic and morphological characters accounted for 78.3% and 60% of the variation, respectively (Fig. 5A, B). In the meristic PCA, the number of total gill rakers, circumpeduncular scales and transverse scale rows below the lateral line loaded heavily on the first PC. Both species were completely distinguished along the first axis (Fig. 5A). In the morphometric PCA, measurements including the length of the soft dorsal-fin base, body depth and depth of the caudal peduncle separated *Plectorhinchus makranensis* sp. nov. from *P. schotaf* along the first PC1 (Fig. 5B).

## Discussion

The present study adds four species (*P. flavomaculatus*, *P. makranensis*, *P. caeruleonothus* and *P. unicolor*) to the previous molecular reconstructions (Sanciangco et al. 2011; Liang et al. 2014; Tavera et al. 2018). Our phylogenetic analysis is consistent with previous morphological and molecular studies. Based on ecological, morphological, color characteristics and biogeography, *Plectorhinchus* is divided into two clades: clade A includes species with deeper body and uniformly dull color compared to clade B (Johnson et al. 2001; McKay 2001; Tavera et al. 2018) (Fig. 2). The species of clade A are usually distributed in the Western Indian Ocean, with two exceptions: *P. macrolepis* and *P. unicolor* are scattered in the Eastern Atlantic and the West Pacific, respectively (Wirtz et al. 2007; Johnson and Wilmer 2015). Species of clade B are usually distributed from the East Indian to the West Pacific Ocean with the exceptions of *P. centurio* and *P. gaterinus*, which are only found in the Western Indian Ocean (Fricke et al. 2018).

A combined morphological and molecular approach should be used to distinguish closely related species (Baldwin et al. 2011; Lavoué and Sullivan 2014; Bogorodsky et al. 2017).

Based on molecular and morphological data (Fig. 2, Suppl. material 2: Table S2), *P. schotaf* is the sister taxon of *P. makranensis* sp. nov. Genetic distance between the new species and *P. schotaf* based on COI and Cyt *b* markers is consistent with species-level divergences in other fish taxa (Johns and Avise 1998; Ward et al. 2005; Hsu et al. 2007; Ward 2009). Also, these two sympatric species in the Persian Gulf and the Gulf of Oman show higher genetic distance than other congeneric species pairs from the East Africa coast to the Red Sea based on both mtDNA markers 3.42% between *P. chubbi* and *P. sordidus* for the COI gene, and 3.50% between *P. chubbi* and *P. sordidus* for the Cyt *b* gene (Suppl. material 1: Table S1).

Because genetic distances between *P. makranensis* sp. nov. and deposited sequences in GenBank for *P. schotaf* and *P. sordidus* (HQ676736, HQ676791, HQ149904, KU499678, KU317896) are low, these deposited specimens could also represent sequences of *P. makranensis* (Fig. 2).

The new species is morphologically most similar to *P. schotaf* and *P. sordidus*. The coloration of the new species differs from *P. schotaf* by having the posterior margin part of the opercular membrane grey (Fig. 4) (vs. red in *P. schotaf* (Fig. 6A, B). The two species also differ in the number circumpeduncular scales 30–31 (vs. 32–34 in *P. schotaf*). Additionally, there are modal differences in counts, transverse scale rows below the lateral line (17–18, modally 17, vs. 18–20, modally 19 in *P. schotaf*), and morphometric differences, with *P. schotaf* having a shorter base of the soft portion of the dorsal fin (Suppl. material 2: Table S2). *Plectorhinchus makranensis* sp. nov. can be distinguished from *P. sordidus* by the number of gill rakers (10–12, modally 12 upper rakers, 16–17, modally 17 lower rakers, 26–29, modally 28, rarely 26 total rakers, vs. 9–11 upper rakers, 15–16 lower rakers, 24–26 total rakers in *P. sordidus*), a longer caudal peduncle and smaller orbit diameter (Suppl. material 2: Table S2) and the first to the third pectoral-fin rays light grey (Fig. 4) (vs. dark grey in *P. sordidus* (Fig. 6C, D)). Additionally, these two species can be different from each other in the number of tubed lateral-line scales (55–57, modally 56, vs. 48–55, modally 54 in *P. sordidus*).

*Plectorhinchus makranensis* sp. nov. is distinguished from other similar congeners as follows: from *P. caeruleonothus* by having 10–12 gill rakers on the upper limb (vs. 7–9 in *P. caeruleonothus*) and 10–11 scales above the lateral line to the base of the first dorsal-fin spine (vs. 15), from *P. unicolor* by having 17–18 transverse scale rows below lateral line (vs. 19–21), from *P. griseus* by having 18–20 dorsal-fin rays (vs. 21–23); from *P. playfairi* in having 55–57 lateral-line tubed scales (vs. 58–60) and 16–17 gill rakers on lower limb (vs. 21–23), from *P. chubbi* by XII dorsal-fin spines and 16–17 gill rakers on the lower limb (vs. XI spines and 21–23 rakers respectively). The number of dorsal-fin spines is XII in new species vs. XIII in *P. chrysotaenia* and XIV in *P. flavomaculatus*, *P. ceylonensis*, *P. gibbosus*, *P. macrolepis* and *P. plagiodesmus*. Furthermore, the ANOVA analysis reveals that the numbers of dorsal-fin spines and soft rays and scales below the lateral line to the first anal-fin spine, as well as the numbers of circumpeduncular scales and total gill rakers, significantly differ from the other examined species. The molecular and morphological differences mentioned above indicate that the new species is separated from other congeners.

## Supplementary Material

XML Treatment for Plectorhinchus makranensis

## Supplementary materials

Supplementary material 1 Table S1 Data type Molecular Explanation note Net Sequence divergence obtained for CO1 (below diagonal) and for Cyt b (above diagonal). https://binary.pensoft.net/file/468317This dataset is made available under the Open Database License (http://opendatacommons.org/licenses/odbl/1.0/). The Open Database License (ODbL) is a license agreement intended to allow users to freely share, modify, and use this Dataset while maintaining this same freedom for others, provided that the original source and author(s) are credited.Ehsan Damadi, Faezeh Yazdani Moghaddam, Fereshteh Ghassemzadeh, Mehdi Ghanbarifardi

Supplementary material 2 Table S2 Data type Morphological Explanation note Meristic and morphometric data for all material examined of *Plectorhinchus makranensis* sp. nov. (N = 16), *P. schotaf* (N = 18) and *P. sordidus* (N = 2) (holotype data for *P. schotaf* and from Smith (1962)). All proportional measurements are given in % of SL and HL. Characters in bold show differences between *P. makranensis* sp. nov. and two other species (p < 0.05). https://binary.pensoft.net/file/468318This dataset is made available under the Open Database License (http://opendatacommons.org/licenses/odbl/1.0/). The Open Database License (ODbL) is a license agreement intended to allow users to freely share, modify, and use this Dataset while maintaining this same freedom for others, provided that the original source and author(s) are credited.Ehsan Damadi, Faezeh Yazdani Moghaddam, Fereshteh Ghassemzadeh, Mehdi Ghanbarifardi
